# Glycated Haemoglobin and Outcomes of Percutaneous Coronary Intervention Among Type Two Diabetic Patients in Saudi Arabia

**DOI:** 10.7759/cureus.11278

**Published:** 2020-10-31

**Authors:** Saad Albugami, Fahad Almehmadi, Ziad M Bukhari, Mohammed S Alqarni, Abdulkarim W Abukhodair, Malak A BinShihon, Faisal Al-Husayni, Razan A Alhazzani, Samah A AlMatrafi, Khalid Makki

**Affiliations:** 1 Cardiology, King Abdullah International Medical Research Center, King Saud Bin Abdulaziz University for Health Sciences, Jeddah, SAU; 2 Cardiology, King Faisal Cardiac Center, King Abdulaziz Medical City, Jeddah, SAU; 3 Medicine, King Saud Bin Abdulaziz University for Health Sciences, Jeddah, SAU; 4 Internal Medicine, King Abdulaziz Medical City, Jeddah, SAU; 5 Cardiac Sciences, King Faisal Cardiac Center, King Abdulaziz Medical City, Jeddah, SAU

**Keywords:** percutaneous coronary intervention, diabetes mellitus, glycated haemoglobin, coronary artery disease, ischemic heart disease, coronary artery intervention, saudi arabia

## Abstract

Background: Glycated haemoglobin (HbA1c) is a marker that reflects the control of diabetes mellitus (DM) over a three-month period. We sought to compare cardiovascular outcomes of diabetic patients with and without controlled levels of HbA1c post percutaneous coronary intervention (PCI) presenting to King Faisal Cardiac Center. Methods: A retrospective single-center study of all patients with type two DM who were treated with PCI during the period between January 2015 and January 2018. All data were obtained from health informatics system. Demographics, clinical data, and major adverse cardiovascular and cerebrovascular events (MACCE) were collected to compare outcomes among diabetic patients with and without controlled HbA1c.

Results: The study included 177 patients with type two DM who underwent PCI. The mean age was 63.3 (SD±12). Males represented 73.4% and 26.6% were females. The mean HbA1c on admission was 8.7%. At presentation 31% of the patients had relatively controlled blood sugar (HbA1c mean 7.5%, SD±0.5) and 69% presented with poorly controlled type two DM (mean HbA1c 9.1%, SD±0.25). The prevalence of hypertension and dyslipidaemia were higher among the uncontrolled group, but there were no differences between both groups in the control of blood pressure or dyslipidaemia. Patients in the uncontrolled group had higher rate of prior PCI (36.6%) compared to the controlled arm (16%, p=0.0195) The prevalence of cerebrovascular, cardiovascular, and renal impairment was similar. The use of insulin was higher among the uncontrolled arm. Patients in the controlled arm had lower incidence of composite endpoints of death and non-fatal myocardial infarction and stroke (MACCE) (14% vs 41%, p=0.001) compared to the uncontrolled arm.

Conclusion: Among patients with type two DM that were treated with PCI, achieving targets of blood sugar control reflected by glycated haemoglobin is associated with improved survival and lower incidence of composite MACCE.

## Introduction

Coronary artery disease (CAD) is one of the deadliest diseases, killing around 7 million people annually [1,2]. Diabetes mellitus (DM) is a strong risk factor for CAD and is usually associated with a higher risk of mortality [1,2]. Diabetes mellitus is a global health issue that affects over 400 million adults worldwide. More than a million of them are in the Kingdom of Saudi Arabia (KSA) [3,4]. A report by the International Diabetes Federation states that in 2015, approximately 5 million diabetics died, and majority of these deaths were due to CAD [3]. Percutaneous coronary intervention (PCI) is widely available as a modality of therapy for patients with symptomatic CAD and many of them are diabetics. Studies show that the number of PCI is rising due to the inevitable trends of an increase in CAD risk factors which include DM [5,6]. Glycaemic control can impact the clinical outcome in diabetic patients after PCI. Several clinical studies found that admission hyperglycaemia (plasma glucose) is associated with increased short- and long-term mortality in diabetic patients with acute myocardial infarction [5-7]. However, acute myocardial infarction is frequently associated with abnormal glucose metabolism leading to hyperglycaemia due to stress-related catecholamines surge. Therefore, serum blood sugar measurements are unstable and unpredictable [7]. Glycated haemoglobin, haemoglobin A1c (HbA1c), is a marker that reflects the control of DM over a three-month period and is hence considered a better prognostic marker of long-term outcome than other glucometabolic parameters reflecting exclusively fasting, postprandial, or incidental glycemia in patients with diabetes [8,9]. HbA1c has been found also to be an independent predictor of in-hospital mortality in ST-elevation myocardial infarction (STEMI) treated with primary PCI [8,9]. It is noteworthy that DM was found to be an independent predictor of mortality in the setting of non-ST-segment elevation acute coronary syndrome. Moreover, DM has been associated with worse outcomes following both percutaneous coronary intervention (PCI) and coronary artery bypass grafting (CABG) [10,11]. In Saudi Arabia, the baseline status, and subsequent results of PCI in diabetic patients with or without controlled glycated haemoglobin have not been well characterized. We aimed to assess the major adverse cardiac and cerebrovascular events (MACCE) among this group of patients who underwent PCI at King Faisal’s Cardiac Center (KFCC) in Jeddah, Saudi Arabia.

## Materials and methods

This study is an observational retrospective cross-sectional study conducted at King Faisal Cardiac Center, Jeddah. The study included all consecutive patients with the confirmed diagnosis of type two DM with available glycated haemoglobin measurements who underwent percutaneous coronary intervention between January 2015 till December 2018. The inclusion criteria were age more than 18 years, treated with PCI, established diagnosis of type two DM, and available HbA1c measurement. Exclusion criteria were CABG as a method of revascularization during index hospitalization, lack of HbA1c measurement on presentation and follow-up, and type one DM.

Patients with available HbA1c at the time of PCI and at six months follow-up were divided into two groups; those with HbA1c <7% at six months follow-up (controlled DM) and those with HbA1c ≥7% at six months follow-up (uncontrolled group). Our endpoints were the accumulative major adverse cardiac and cerebrovascular events, defined as death, nonfatal myocardial infarction (clinical presentation with acute coronary syndrome) and stroke. The data were collected from patients ‘electronic files, including gender, age, date of procedure, medical history, laboratory values, pharmacological therapy and interventional treatment during index hospitalization and follow-up. The study was conducted following the Declaration of Helsinki. It received ethical approval from the Institutional Review Board of King Abdullah International Medical Research Center number IRBC/0266/19.

Statistical analysis

Participant’s baseline characteristics were presented as means and standard deviations for continuous covariates, and as proportions for categorical covariates. Continuous covariates compared using two-sample t-test assuming normality of distributions under the Central Limit Theorem where categorical ones compared using Fisher’s exact test. Time-to-event analysis for patients with controlled HbA1c (at follow-up <7%) to those who were uncontrolled (HbA1c ≥7%) was performed using log-rank test. Univariate Cox-regression performed to identify predictors of MACCE, and any variables with p-value of <0.1 (namely; HbA1c ≥7%, active smoking, use of dipeptidyl peptidase 4 [DPP-4] inhibitors, use of metformin, insulin use, total cholesterol level and body mass index) added to a multivariate model. Except for univariate cox-regression covariate exploration, a two-sided p-value of <0.05 was considered to be significant. All statistical analyses were performed using R software version 4.0.0 (R Foundation for Statistical Computing, Vienna, Austria) [12].

## Results

During the study period, 1218 patients had PCI and only 177 patients with type two DM had available glycated haemoglobin during index hospitalization and follow-up. The mean age was 63.2 years (SD±12). Among the controlled group the mean age was 60.82 years (SD±13.03) while in the uncontrolled arm was 64.3 (SD±11.33) this was not significant (p=0.089). The majority of patients were men (73.4%). The mean glycated haemoglobin on presentation for the entire study patients was 8.7% (SD±1.9). In the controlled group the mean HbA1c was 7.56% (SD±0.5) while among the uncontrolled group it was 9.19% (SD±0.25, p<0.001).The uncontrolled group had significantly higher number of patients with hypertension (86.9% vs 63.4%, p<0.00097), dyslipidemia (69.7% vs 47.3%, p<0.0068) and prior coronary intervention (36.6% vs 16.4%, p<0.0195) though there were no significant differences in other associated conditions like smoking, prior stroke or obesity. The level of blood pressure and dyslipidaemia control was similar between both groups. Mean systolic blood pressure was 129 mmHg (SD±24) vs 133 mmHg (SD±19), p=0.363, while the low-density lipoprotein was 2.38 mmole/dl (SD±0.89) vs 2.41 mmole/dl (SD±0.98), p=0.818. With regard to the pharmacological agents during the study period, there were no significant differences between both groups except for significantly higher use of insulin among the uncontrolled arm (68% vs 30.9%, p<0.0001) (Table 1).

**Table 1:** Demographics of the participants. ●    Data are presented as Mean (Standard Deviation) for continuous variables or in proportions (%) for categorical variables. ●    Values were missing in two patients (*) for some covariates and three patients (§) for others. HbA1c = haemoglobin A1c, ACEI = angiotensin-converting enzyme inhibitors, ARB = angiotensin receptor blocker

Variable	Overall N=177	HbA1C at Follow up		P-value
		≤7 %	>7 %	
		N=55	N=122	
Demographic Data				
Age in years	63.2 (12)	60.82(13.03)	64.3 (11.33)	NS 0.089
Male sex	130 (73.4)	44(80)	86(70.5)	NS 0.203
Medical Comorbidities				
HbA1C level in % at admission	8.7 (1.9)	7.56 (0.5)	9.19(0.25)	<0.001
HbA1C level (change in follow up) in %	-0.03 (1.87)	-0.78 (1.87)	0.29(1.78)	<0.001
Hypertension	141(79.9)	35(63.4	106(86.9)	<0.00097
Dyslipidaemia	111(62.7)	25(47.3)	85(69.7)	0.0068
Active smoking	34 (19.2)	14(25.5)	20(16.4)	NS 0.215
Body mass index in	25.1 (4.51)	25.11(4.3)	25.12(4.6)	NS 0.987
Prior Coronary intervention	50 (28.2)	9(16.4)	41(36.6)	0.0195
Prior Coronary artery bypass grafting	14(7.9)	2(3.6)	12(9.8)	NS 0.607
Documented three vessel disease	7(4)	1(1.8)	6(4.9)	NS 0.32
Prior stroke	8(4.5)	2(3.6)	6(4.9)	NS 0.687
Clinical Variables				
Systolic blood Pressure (mmHg)	132.4(20	129(24)	133(19)	NS 0.363
17 Left Ventricular Ejection Fraction (%)	47.7(9.4)	49.5(9.4)	46.9(9.4)	NS 0.089
Creatinine Clearance, (mL/min/1.73 m2)	74.59(39)	80(37)	72(40)	NS 0.204
Total Cholesterol	4.21(1.2)	4.02(1.2)	4.29(1.2)	NS 0.1614
Low-Density-lipoprotein (LDL) cholesterol	2.4(0.94)	2.38(0.89)	2.41(0.98)	NS 0.818
High-Density-lipoprotein (HDL) cholesterol	0.91(0.23)	0.852(0.214)	0.934(0.231)	0.025
Medications				
Aspirin *	174 (98.9)	55(100)	119(97.5)	NS 1
Minimum of 1 year of P2Y12 inhibitor*	174 (98.9)	54(98.2)	116(95.1)	NS 1
High-dose Statin§	161(92)	49(89.1)	112(91.8)	NS 0.315
ACEI / ARB§	141(80.6)	40(72.2)	101(82.2)	NS 0.0936
Beta-blocker§	148(84.6)	48(81.8)	103(84.4)	NS 0.5
Mineralocorticoid inhibitor§	24 (13.7)	8(14.5)	16(13.1)	NS 0.797
Sacubitril /Valsartan§	1(<1)	1(1.8)	0(0)	NS 1
Anti-diabetic modalities				
Metformin§	104(59.4)	37(67.3)	67(54.9)	NS 0.19
Sulfonylurea §	41(54.7)	12(21.8)	30(24.6)	NS 0.6086
Insulin use §	100(57.1)	17(30.9)	83(68)	<0.0001
Dipeptidyl Peptidase-4 (DPP-4) Inhibitor§	39(22.3)	7(12.7)	32(26.2)	NS 0.07319
Thiazolidinedione-type (TZD) §	1(<1)	0(0)	1(<1)	NS 1
Acylated Glucagon-Like Peptide-1 (GLP-1) agonist	7(3.9)	2(3.6)	5(4.1)	NS1

The composite endpoints of refractory angina, acute coronary syndrome, all-cause cardiovascular death and stroke was significantly higher among the uncontrolled arm (14% vs 41.8%, p<0.00036) (Table 2).

**Table 2:** Demonstrates the adverse cardiac and cerebrovascular events DM = diabetes mellitus

	Controlled DM N= 55	Uncontrolled DM N=122	P value
Refractory angina	3 (5.4%)	18 (14.7 %)	
Acute Coronary Syndrome	4 (7.2%)	25 (20.49%)	-
Sudden Death	0	3 (2.4%)	-
Cardiovascular mortality	1 (1.8%)	3 (2.4%)	-
Stroke	0	2 (1.6%)	-
Composite end points	8 (14%)	51 (41.8%)	0.00036

Table 3 shows univariate Cox-regression analysis of covariates in predicting major cardiovascular events. On the analysis, factors that were associated with increased major adverse cardiovascular events (MACCE) were glycated haemoglobin level (odds ratio (OR) 2.96, 95% confidence interval (CI) (1.5-5.86), p=0.00177). Active smoking status, body mass index, cholesterol level, use of insulin, metformin and DPP-4 inhibitors. While on multivariate analysis glycated haemoglobin level was the only predictor of higher MACCE (OR 2.98, 95% CI (1.44-6.17), p=0.00321) in addition to active smoking (OR 1.8937, 95% CI (1.021-3.512), p=0.0428) as demonstrated in Table 4.

**Table 3:** Univariate Cox-regression analysis of covariates in predicting major cardiovascular events. * Covariates with p-value ≤ 0.1 included in multivariate analysis. § We chose the follow-up HbA1c as it is more indicative of medical therapy adequacy after the index event. HbA1c = haemoglobin A1c, ACEI = angiotensin-converting enzyme inhibitors, ARB = angiotensin receptor blocker

Covariate	Hazard Ratio	Lower CI limit	Upper CI limit	p-value
HbA1C at entry	2.16	1.06	4.41	0.0345
HbA1C during follow up	2.96	1.5	5.86	0.00177*§
Change in HBA1C in follow up	1.05	0.91	1.207	0.513
Age in years	1	0.98	1.025	0.774
Body mass index	0.94	0.89	1.007	0.0788*
Dyslipidaemia	1.4	0.86	2.59	0.151
Hypertension	1.75	0.8	3.6	0.142
Prior coronary stenting	1.007	0.574	1.766	0.982
Prior Coronary bypass	1.306	0.5598	3.04	0.536
Prior Acute Coronary Syndrome	1.525	0.759	3.015	0.245
Active smoking	1.716	0.953	3.09	0.0722*
Presence of 3 vessel disease	1.56	0.488	4.99	0.452
Left Ventricular Ejection Fraction	0.99	0.98	1.02	0.60
Creatinine Clearance	0.944	0.986	1.00	0.17
Low-Density-lipoprotein (LDL) cholesterol	1.159	0.8815	1.524	0.291
High-Density-lipoprotein (HDL) cholesterol	1.23	0.39	3.85	0.723
Total Cholesterol	1.209	0.984	1.1484	0.072*
Use of ACEI/ ARB	1.311	0.6	2.67	0.456
Use of High dose Statin	1.484	0.527	4.176	0.445
Use of Dipeptidyl Peptidase-4 (DPP-4) Inhibitor	0.536	0.26	1.093	0.086*
Use of Sulfonylurea	0.7484	0.388	1.443	0.387
Insulin use	1.59	0.92	2.755	0.092*
Use of Metformin	0.505	0.3012	0.8467	0.00956*

**Table 4:** Multivariate Cox-regression analysis of covariates in predicting major cardiovascular events. (Final model included the following covariates: HbA1c at follow-up, use of insulin, use of DPP-4 inhibitor, use of metformin, BMI, total cholesterol, and active smoking.) HbA1c = haemoglobin A1c

Covariate	Hazard Ratio	Lower CI limit	Upper CI limit	p-value
HbA1C >7% at follow-up	2.98	1.44	6.17	0.00321*
Use of Dipeptidyl Peptidase-4 (DPP-4) Inhibitor	0.55	0.26	1.14	0.108
Insulin use	1.06	0.57	1.97	0.853
Use of Metformin	0.65	0.365	1.15	0.138
Total Cholesterol	1.13	0.91	1.42	0.272
Active smoking	1.82	0.981	3.38	0.0577
Body mass index (BMI)	0.96	0.91	1.03	0.245

**Figure 1:**
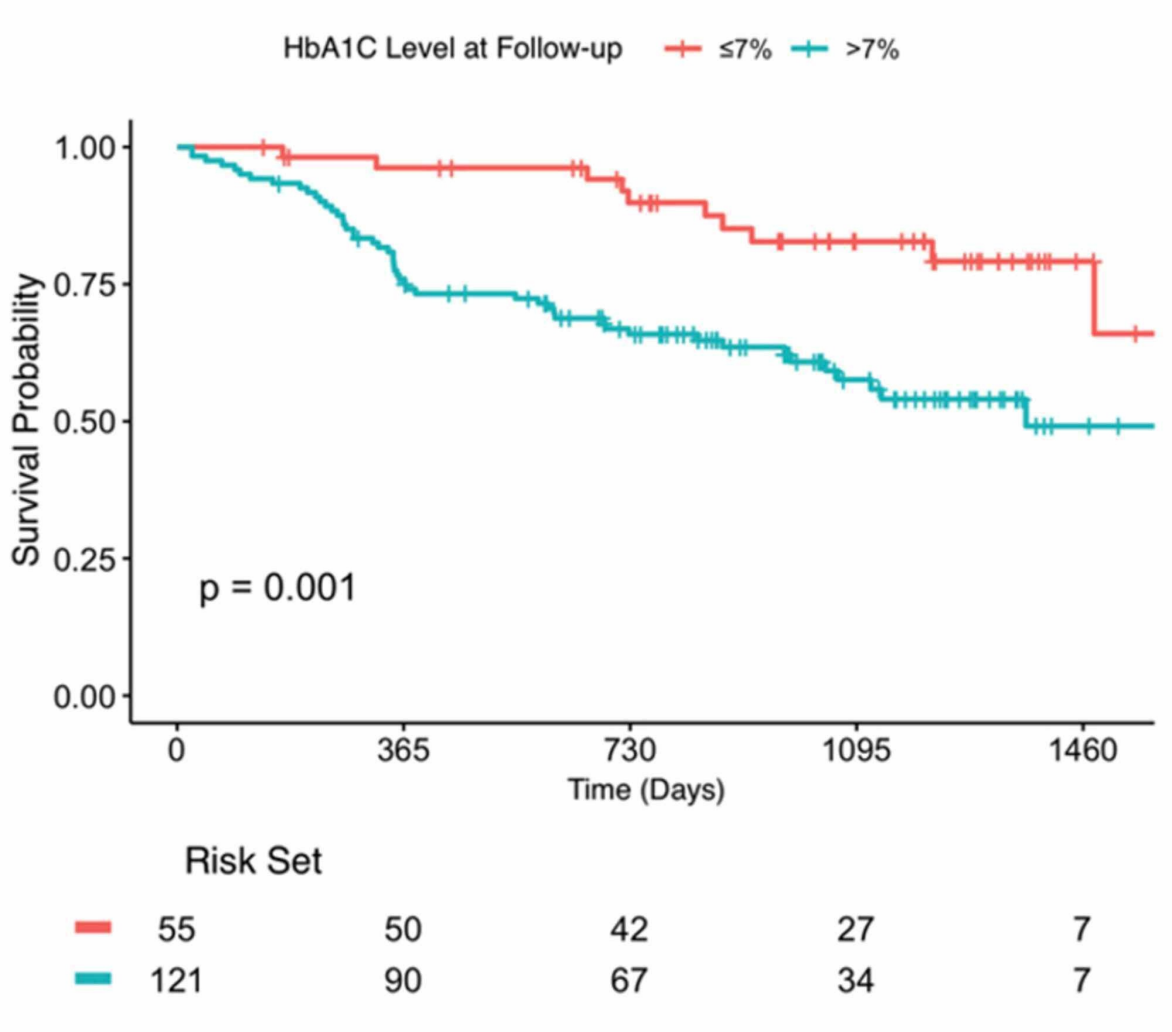
Kaplan-Meier curves showing event-free survival of each HbA1c. HbA1c = haemoglobin A1c

Figure 1 shows the Kaplan Mayer curves which demonstrates the effects of higher glycated haemoglobin post-PCI and the increased mortality that was associated with poor HbA1c control.

**Figure 2:**
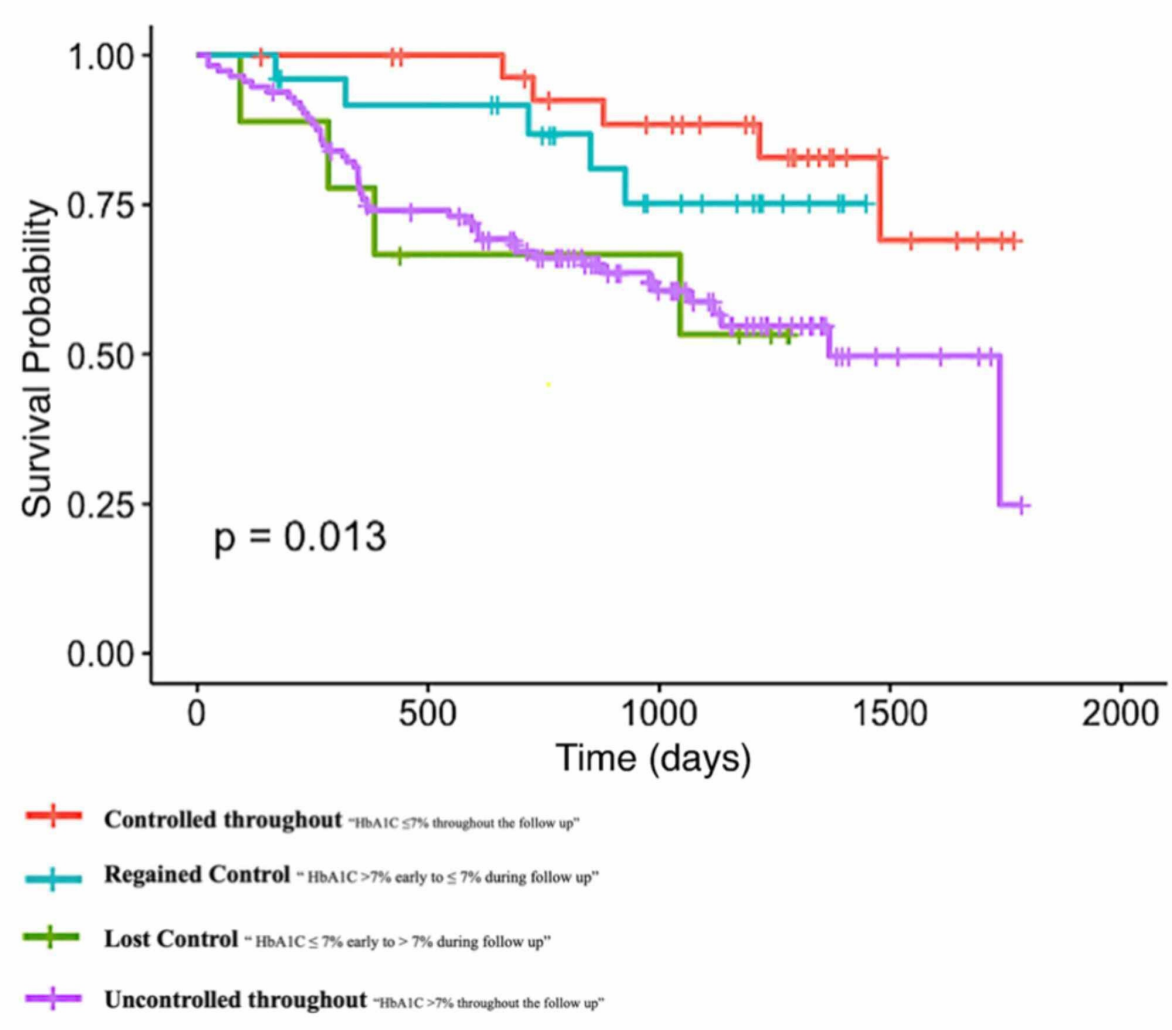
Kaplan-Meier curves showing event-free survival probability based on the change of HbA1c status during follow-up period. HbA1c = haemoglobin A1c

Patients who maintain or achieve guideline recommended HbA1c of less than 7% have improved survival while those who either continue to have high levels or lose adequate control and or never achieve HbA1c of less than 7% at follow up have higher rate of mortality (Figure 2).

## Discussion

The overall prevalence of CAD as assessed by various diagnostic methods is as high as 55% among adult patients with DM, compared with 2 to 4% in the general population [13-18]. It is considered an independent risk factor for increased mortality and morbidity [13-18]. The cardiovascular mortality rate is more than doubled in men and more than quadrupled in women who have DM, compared with their non-diabetic counterparts [14-15]. DM is a recognized risk factor for poor outcome following either percutaneous coronary or surgical revascularization [15-18]. In Saudi Arabia, the prevalence of diabetes among ACS patients is 56% as reported previously [19,20]. Those patients are more likely to present with non-ST-elevation myocardial infarction (NSTEMI) or unstable angina compared to non-diabetic patients (40.2% vs 31.4%, and 22.6% vs 21.1%, respectively), and less likely to present with STEMI (37.2% vs 47.5%; p=0.001) as reported by AlNemer et al. In addition, they have a higher rate of major adverse cardiovascular outcomes [21].

Many randomized clinical trials have demonstrated that therapies that improve glycaemic control decrease the risk of microvascular disease, including retinopathy, nephropathy, and neuropathy [22]. However, trials attempting to decrease macrovascular events have been unsuccessful [23-25], improved glycaemic control showed no reduction in the rate of cardiovascular events and in the Action to Control Cardiovascular Risk in Diabetes (ACCORD) trial [23], it was associated with increased risks of death from any cause and death from cardiovascular events.

A breakthrough in the management of patients with type two DM at high risk for cardiovascular events was reported by Zinman and colleagues [26] with the use of empagliflozin which demonstrated a reduction in the rate of the primary composite cardiovascular outcome and of death from any cause when the study drug was added to standard care. The adjusted mean differences in the glycated haemoglobin level between patients receiving empagliflozin and those receiving placebo were −0.54 percentage points (95% CI, −0.58 to −0.49) in the 10 mg group and −0.60 percentage points (95% CI, −0.64 to −0.55) in the 25 mg group, although many patients did not reach their glycaemic targets, with an adjusted mean glycated haemoglobin level at week 206 of 7.81% in the pooled empagliflozin group and 8.16% in the placebo group. 

Our single-center retrospective study has demonstrated the favourable effect of maintaining and achieving the guideline-recommended level of HbA1c in diabetic patients post-PCI. The composite endpoints of MACCE were statistically better among patients with controlled HbA1c levels at follow-up. Although there were statistically significant differences between both groups as the uncontrolled arm had a higher rate of hypertension and dyslipidaemia, there was however no difference in the level of control of these two risk factors, the facts that the uncontrolled group had a higher rate of use of medication, especially insulin, would suggest the heightened efforts to control DM or might reflect advanced DM.

The results of our study are consistent with what was previously reported by Kessaian and colleagues [27] where they have reported that diabetics with poor glycaemic control based on levels of HbA1c are at two-fold higher risk of developing MACCE following PCI. Other investigators [8-28] have also observed lower rates of target vessel revascularization (TVR), cardiac rehospitalization and recurrent angina in optimally controlled (HbA1c ≤7%) in diabetic patients.

However other studies have examined the potential association between HbA1c and clinical outcomes among DM patients after PCI, these studies were inconclusive and sometimes even contradictory, due to the fact that the majority of these studies were small-sized, leading to inadequate statistical power. A recent large meta-analysis by Zheng et al. [29] demonstrated that high blood HbA1c levels might be associated with higher risks of target vessel revascularization, progression of CAD, and nonfatal myocardial infarction among diabetic patients after PCI. We have noted that other factors can be significantly associated with increased MACCE among diabetic patients, namely smoking, obesity, use of insulin, and DDP-4 inhibitors. When controlling for other variables, active smoking and HbA1c were the only predictors for increased MACCE in our study.

To the best of our knowledge, this is the first study in the Saudi population that assessed the post-procedural impact of HbA1c control following PCI and MACCE. A large, prospective, and multicentre study is underway.

Limitations

Our study has many limitations, it is retrospective therefore it has an inherent selection bias as many patients who were treated by PCI were excluded due to lack of HbA1c level on presentation and follow-up. It's a single-center study, which may affect generalizability of its conclusion. We also did not assess the duration of DM and the indications for revascularization whether this is done due to an acute coronary syndrome or as an elective procedure for chronic angina. Another limitation is the small number of patients. Yet the results provided reflect data consistent with other studies linking poor outcomes of patients with coronary artery disease and uncontrolled DM.

## Conclusions

Patients with coronary artery disease and uncontrolled DM reflected by HbA1c above 7% at or during follow-up have an increased rate of death and non-fatal MI compared to patients who maintain or achieve guidelines recommended HbA1c of less than 7%. Large and multicentre studies are required to further ascertain this finding. Guidelines recommending control of type two DM will favourably improve long-term outcomes of coronary artery disease.
